# Immunohistological Analysis of the Jun Family and the Signal Transducers and Activators of Transcription in Thymus

**DOI:** 10.1155/2015/541582

**Published:** 2015-03-18

**Authors:** Alexandra Papoudou-Bai, Alexandra Barbouti, Vassiliki Galani, Kalliopi Stefanaki, Panagiotis Kanavaros

**Affiliations:** ^1^Department of Pathology, University of Ioannina, 45110 Ioannina, Greece; ^2^Department of Anatomy-Histology-Embryology, University of Ioannina, 45110 Ioannina, Greece; ^3^Department of Pathology, Agia Sofia Children's Hospital of Athens, 11527 Athens, Greece

## Abstract

The Jun family and the signal transducers and activators of transcription (STAT) are involved in proliferation and apoptosis. Moreover, c-Jun and STAT3 cooperate to regulate apoptosis. Therefore, we used double immunostaining to investigate the immunotopographical distribution of phospho-c-Jun (p-c-Jun), JunB, JunD, p-STAT3, p-STAT5, and p-STAT6 in human thymus. JunD was frequently expressed by thymocytes with higher expression in medullary compared to cortical thymocytes. p-c-Jun was frequently expressed by cortical and medullary thymic epithelial cells (TEC) and Hassall bodies (HB). p-STAT3 was frequently expressed by TEC with higher expression in cortical compared to medullary TEC and HB. p-c-Jun, JunB, p-STAT3, p-STAT5, and p-STAT6 were rarely expressed by thymocytes. JunB and JunD were expressed by rare cortical TEC with higher expression in medullary TEC. p-STAT5 and p-STAT6 were expressed by rare cortical and medullary TEC. Double immunostaining revealed p-c-Jun and JunD expression in rare CD11c positive dendritic cells. Our findings suggest a notable implication of JunD in the physiology of thymocytes and p-c-Jun and p-STAT3 in the physiology of TEC. The diversity of the immunotopographical distribution and the expression levels of p-c-Jun, JunB, JunD, p-STAT3, p-STAT5, and p-STAT6 indicates that they are differentially involved in the differentiation of TEC, thymocytes, and dendritic cells.

## 1. Introduction

The activator protein-1 (AP-1) is a dimeric transcription factor that contains members from the Jun (c-Jun, JunB, and JunD), Fos (c-Fos, FosB, Fra-1, and Fra-2), and activating transcription factor (ATF) protein families [1–4]. AP-1 is involved in cell differentiation, proliferation, survival, and apoptosis [1–4]. AP-1 activity is regulated by a wide variety of stimuli including cytokines and growth factors which activate mitogen-activated protein kinase (MAPK) cascades [1, 2]. Extracellular signal-regulated kinase (ERK), c-Jun N-terminal kinase (JNK), and the p38 MAP kinases phosphorylate specific substrates of the AP-1 family with JNK regulating both c-jun and junB phosphorylation [1, 2, 5]. C-Jun generally promotes cell proliferation through induction of CCND1 (cyclin D1) and inhibition of p16, p21, and p53 [1]. JunB may inhibit cell proliferation through both positive effects on genes such as the p16 and negative effects on genes such as cyclin D1 during the G1-phase [1, 2]. JunB may also promote a cell-division-promoting activity, in particular, via stimulation of cyclin A2 gene expression during S-phase [1, 2]. JunD may be a positive or negative regulator of cell proliferation in a context-dependent manner and may protect cells from oxidative stress [2, 4].

Interestingly, there is evidence that AP-1 may interact with other transcription factors such as signal transducer and activator of transcription 3 (STAT3) [6]. The STAT proteins (STAT1, STAT2, STAT3, STAT4, STAT5A/B, and STAT6) are involved in cell differentiation, proliferation, survival, and apoptosis and can be activated via the Janus kinases- (JAK-) STAT pathway [7–9]. The interaction of a wide variety of cytokines and growth factors with their cognate receptors leads to JAK activation, which in turn phosphorylates and activates STAT; the activated STAT then translocate to the nucleus to regulate their target genes [8]. The transcriptional targets of STAT proteins play roles in cell cycle progression (e.g., cyclin D1, cyclin D3, and p21) and survival (e.g., bcl-xL, mcl-1, and bcl-2) [7–9]. Interestingly, there is evidence indicating that AP-1 and JAK-STAT are involved in thymic cell physiology [10–16].

The human thymus supports the production of self-tolerant T-cells with competent and regulatory functions and their migration to the peripheral blood circulation [17, 18]. The thymus contains other various cells such as thymic epithelial cells (TEC) and dendritic cells and reciprocal interactions between these cells and thymocytes play essential roles in thymic physiology [19–27]. Interestingly, the multiple cellular events occurring during thymic T-cell (thymocyte) and TEC differentiation involve major signaling pathways regulating cell proliferation, survival, and apoptosis [17–19, 21–25]. In this respect, immunohistological studies permitting the identification of human thymic cells expressing various elements of major signaling pathways such as the MAPK and the JAK-STAT pathways provided information for the understanding of thymic histophysiology [28–30]. For example, immunohistological analysis of the MAPK pathway in 11 cases of human thymuses from newborns and children showed that (a) p-ERK was abundantly present in the outer layer of HB, (b) p-p38 kinase was present in the entire HB, and (c) p-JNK was expressed in medullary thymocytes [30]. Furthermore, immunohistological analysis of the JAK-STAT pathway in the same 11 cases of human thymuses [30] showed that (a) JAK1, JAK2, and TYK2 were expressed in high amounts in the entirety of HB, whereas JAK3 was expressed in the outer layer of HB and (b) STAT1, STAT2, and STAT6 were abundantly expressed in the entire HB, whereas STAT5 was expressed in the outer layer of HB [29].

Despite evidence indicating that (a) JAK-STAT and AP-1 are involved in thymic cell physiology and pathology [10–16, 29–33], (b) cooperation between STAT3 and c-Jun may be involved in the regulation of apoptosis [6], and (c) JunD modulates the expression of the suppressor of cytokine signaling 1 (SOCS1) which is a negative regulator of JAK-STAT pathway [34, 35], to the best of our knowledge, detailed immunohistological analysis of the Jun family and p-STAT proteins in normal human thymic tissue has not been reported so far. This analysis may provide useful information for further understanding not only of the thymic histophysiology but also of the thymic tumor pathology since Jun and STAT proteins are implicated in the pathogenesis of various tumors (reviewed in [2, 7–9]). Therefore, we used double immunostaining on the same tissue section with markers of epithelial cells, lymphoid cells, dendritic cells, and macrophages in order to determine the (a) topographical distribution and (b) the expression levels of p-c-Jun, JunB, JunD, p-STAT3, p-STAT5, and p-STAT6 in human thymuses. These thymuses have been analyzed in our previous studies for the immunohistological expression of various cell differentiation markers and proteins involved in the regulation of cell proliferation and apoptosis [36–38]. The findings of our present study suggest a notable implication of JunD in the physiology of thymocytes and p-c-Jun and p-STAT3 in the physiology of TEC.

## 2. Materials and Methods

### 2.1. Material

The material of the present retrospective study consisted in paraffin blocks of formalin-fixed thymuses which were retrieved from the files of the Department of Pathology, Agia Sofia Children's Hospital of Athens. Paraffin sections from 10 histologically normal postnatal human thymuses (3 newborns and 7 children) were included in the present study. The thymic tissues of the present retrospective study were obtained from newborns and children who underwent corrective cardiovascular surgery and the removal of the thymic tissue was required to expose the operative site. Some of the thymuses of the present study were analyzed in our previous studies for the immunohistological expression of cytokeratins, neural/neuroendocrine markers, markers of dendritic cells, markers of macrophages, and proteins involved in cell proliferation and apoptosis regulation [36–38]. This retrospective study was approved by the Scientific Board of the University Hospital of Ioannina (No 8751).

### 2.2. Methods

The streptavidin-biotin peroxidase-labeled (LSAB) procedure, the alkaline phosphatase/anti-alkaline phosphatase (APAAP), and the double (on the same section) LSAB/APAAP immunohistochemical procedures were used on formalin-fixed and paraffin-embedded tissue sections. The following antibodies were used: p-c-Jun (polyclonal, Ser73, Cell Signaling, dilution 1 : 30), JunB (monoclonal, C-11, Santa Cruz Biotechnology, dilution 1 : 70), JunD (polyclonal, SC-74, Santa Cruz Biotechnology, dilution 1 : 100), p-STAT3 (polyclonal, Tyr705, Cell Signaling, dilution 1 : 300), p-STAT5 (polyclonal, Tyr694, Cell Signaling, dilution 1 : 50), and p-STAT6 (polyclonal, Tyr641, Cell Signaling, dilution 1 : 50). Positive control slides such as reactive lymph nodes and diffuse large B-cell non-Hodgkin's lymphomas from our previous study [39] were included in the present study. The immunostainings were analyzed by light microscopy using a Nikon eclipse 50i microscope. Cells showing positive immunostaining with LSAB and APAAP were labeled brown and red, respectively. The percentage of immunopositive cells was evaluated independently by two observers. The evaluation of immunostaining and the definition of groups of positivity were based on our previous studies [36–38, 40]. Briefly, a continuous score system was adopted by using the ×40 objective lens and by counting the immunopositive cells in ten fields per section (five in the cortex and five in the medulla). The number of immunopositive cells was divided by the total number of the counted cells and the expression of each protein in each case was defined as the percentage of positive cells in the total number of the cells counted. Then, the percentages of positivity of each protein in each case were allocated to groups of positivity which were determined as range of percentages using cut-off levels as follows: less than 1% (−/+); 1–10% (+/−); 10–20% (+); 20%–50% (++); and more than 50% (+++). In order to achieve a better identification of cells expressing the proteins p-c-Jun, JunB, JunD, p-STAT3, p-STAT5, and p-STAT6, besides morphological criteria, we used our previously reported double immunostaining approach on the same histological section with a panel of antibodies (pan-cytokeratin for epithelial cells, chromogranin for cells with neuroendocrine differentiation, CD3 for T-cells, CD20 for B-cells, S-100 protein, CD11c, CD123 and CD207 for dendritic cells, CD163 for macrophages, desmin for myoid cells, CD34 for endothelial cells, and smooth muscle actin for vascular smooth muscle) [36–38, 40]. The Hassall bodies (HB) were classified as juvenile, immature, mature, senescent, and lymphocyte-rich subtypes according to previous studies [41, 42]. Briefly, juvenile HB were small-sized or medium-sized, ovoid or irregular formations of thymic epithelial cells with unaltered cellular morphology, without prominent cytoplasmic acidophilia, necrosis or cellular debris. Immature HB were round or oval formations, consisting of squamous thymic epithelial cells with cytoplasmic acidophilia, but without degenerative changes. Mature HB were medium- sized or large-sized epithelial formations, with necrotic or cystic degenerative changes in their central area, but presenting squamous thymic epithelial cells at their periphery. Senescent HB were large-sized formations, without any morphologically recognizable thymic epithelial cells, but with calcified, necrotic material, cellular debris, or cystic dilatation. Lymphocyte-rich HB were medium- or large-sized formations, containing groups of lymphocytes [41, 42]. The counting of immunopositive cells was performed in all subtypes of HB except for senescent HB [42].

### 2.3. Statistical Analysis

Student's *t*-test was used for statistical analysis. The results were considered as statistically significant when *P* < 0.05. The program SPSS Statistics Release 20 was used for statistical analysis.

## 3. Results

### 3.1. Immunohistological Expression Patterns of p-c-Jun, JunB, JunD, p-STAT3, p-STAT5, and p-STAT6 in Thymocytes (Table 1 and Figures 1 and 2)

p-c-Jun, JunB, p-STAT3, p-STAT5, and p-STAT6 nuclear expression was detected in a few scattered cortical and medullary thymocytes (<1%).

JunD nuclear expression was detected in cortical and medullary thymocytes. Statistical analysis showed that the expression levels of JunD were significantly higher in medullary (mean value 31.20% ± standard deviation 5.41%) compared to cortical thymocytes (mean value 6.60% ± standard deviation 1.71%) (*t*-test, *P* < 0.001).

### 3.2. Immunohistological Expression Patterns of p-c-Jun, JunB, JunD, p-STAT3, p-STAT5, and p-STAT6 in Thymic Epithelial Cells (Table 2 and Figures 1 and 2)

p-c-Jun nuclear expression was detected in cortical (subcapsular and inner cortical) and medullary TEC and in cells of the outer layers of HB, mainly of the juvenile and immature subtypes. p-c-Jun immunoreactive subcapsular TEC outnumbered p-c-Jun immunoreactive inner cortical TEC. Statistical analysis did not show significant differences between the expression levels of p-c-Jun in cortical TEC (subcapsular and inner cortical) (mean value 30.70% ± standard deviation 5.31%), medullary TEC (mean value 32.30% ± standard deviation 5.61%), and HB (mean value 26% ± standard deviation 4.21%).

JunB nuclear expression was detected in cortical (subcapsular and inner cortical) and medullary TEC and in cells of the outer layers of HB, mainly of the juvenile and immature subtypes. Statistical analysis showed that the expression levels of JunB were significantly higher in medullary (mean value 6.30% ± standard deviation 1.70%) compared to cortical TEC (<1%) (*t*-test, *P* < 0.001) and in HB (mean value 3.60% ± standard deviation 1.17%) compared to cortical TEC (<1%) (*t*-test, *P* < 0.001).

JunD nuclear expression was detected in cortical (subcapsular and inner cortical) and medullary TEC and in cells of the outer layers of some HB, mainly of the juvenile and immature subtypes. Statistical analysis showed that the expression levels of JunD were significantly higher in medullary (mean value 11.90% ± standard deviation 1.52%) compared to cortical TEC (<1%) (*t*-test, *P* < 0.001) and in HB (mean value 11.70% ± standard deviation 1.25%) compared to cortical TEC (<1%) (*t*-test, *P* < 0.001).

p-STAT3 nuclear expression was detected in cortical (subcapsular and inner cortical) and medullary TEC and in cells of the outer layers of some HB, mainly of the juvenile and immature subtypes. p-STAT3 immunoreactive subcapsular TEC outnumbered p-STAT3 immunoreactive inner cortical TEC. Statistical analysis showed that the expression levels of p-STAT3 were higher in cortical (subcapsular and inner cortical) (mean value 30.50% ± standard deviation 6.22%) compared to medullary TEC (<1%) (*t*-test, *P* < 0.001) and HB (<1%) (*t*-test, *P* < 0.001).

p-STAT5 and p-STAT6 nuclear expression were detected in rare scattered medullary TEC (<1%) and in rare cells in the outer layers of a few HB, mainly of the juvenile and immature subtypes (<1%). Some endothelial cells exhibited p-c-Jun, p-STAT3, and p-STAT5 nuclear expression. Some vascular muscle cells exhibited p-c-Jun and p-STAT3 nuclear expression.

### 3.3. Results of Double Immunostainings (Figures 1 and 2)

We first analyzed the immunostaining pattern of cell-differentiation markers (pan-cytokeratin MNF116 for epithelial cells, chromogranin for cells with neuroendocrine differentiation, CD3 for T-cells, CD20 for B-cells, S-100 protein, CD11c, CD123, and CD207 for dendritic cells, CD163 for macrophages, desmin for myoid cells, CD34 for endothelial cells, and smooth muscle actin for vascular smooth muscle) which were used in double immunostaining experiments in order to achieve a better identification of cells expressing p-c-Jun, JunB, JunD, p-STAT3, p-STAT5, and p-STAT6. Pan-cytokeratin MNF116 staining showed a lacy network of TEC in cortex and a more condensed TEC network in medulla. A few chromogranin immunoreactive cells were detected, mainly localized in the medulla and HB mainly of the juvenile and immature subtypes. CD3 immunoreactive cells were numerous in both cortex and medulla. CD20 immunoreactive cells were mainly localized in the medulla with an obvious tendency to concentrate around HB. S100 immunoreactive cells were found in medulla and HB, mainly of the juvenile and immature subtypes. CD207 immunoreactive cells were identified in medulla and HB, mainly of the juvenile and immature subtypes. CD11c immunoreactive cells were detected in inner cortex, medulla, and HB, mainly of the juvenile and immature subtypes. CD123 immunoreactive cells were observed in medulla. CD163 immunoreactive cells were found in inner cortex, medulla, and HB. A few desmin immunoreactive cells were detected in medulla nearby HB, mainly of the juvenile and immature subtypes. CD34 and smooth muscle actin stained endothelial cells and vascular smooth muscle cells, respectively. p-c-Jun, JunB, JunD, and p-STAT3 nuclear expression was detected in some pan-cytokeratin MNF116 positive cells (epithelial cells). p-c-Jun and JunD nuclear expression was observed in a few CD11c positive cells (dendritic cells). p-c-Jun, JunB, and JunD nuclear expression was observed in some CD3 positive cells (T-cells). No double immunostaining in thymic cells was observed using the following combinations of antibodies: CD20, S-100, CD123, CD207, CD163, or chromogranin with p-c-Jun, JunB, JunD, or p-STAT3.

## 4. Discussion

The Jun family and the STAT proteins play key roles in cell differentiation, proliferation, and apoptosis. Moreover, cooperation between c-Jun and STAT3 may be involved in the regulation of apoptosis [6]. Although there is a body of evidence indicating that AP-1 and JAK-STAT are involved in thymic cell physiology and pathology [10–16, 29–33], to the best of our knowledge, detailed immunohistological analysis of the expression patterns of the Jun family and p-STAT proteins in normal thymic tissue has not been reported so far. Therefore, we used double immunostainings on the same tissue section in order to determine (a) the topographical distribution and (b) the expression levels of p-c-Jun, JunB, JunD, p-STAT3, p-STAT5, and p-STAT6 in histologically normal postnatal human thymus.

In the present study, p-c-Jun was expressed by a sizeable proportion of cortical and medullary TEC including cells in HB. Since c-Jun generally promotes cell proliferation, these findings suggest that activation (phosphorylation) of c-Jun may have notable involvement in the positive regulation of TEC proliferation [2]. On the other hand, our findings that the expression levels of JunB and JunD are low in cortical and higher in medullary TEC (including HB) are consistent with evidence that JunB and JunD are positive regulators of cell maturation in several cell types such as keratinocytes, osteoblasts, and spermatocytes [1, 2, 4]. Furthermore, c-Jun and JunB may also be involved in the regulation of autophagy which is an important function of TEC, implicated in thymic antigen presentation and T-cell education [10, 26, 27]. It should be noticed that the relationship between Jun family and proliferation of TEC cannot be clearly analyzed only on immunohistological grounds since both cortical and medullary TEC exhibit low proliferation profile (determined by immunostaining with Ki67 and cyclins A, B1, D3, and E) [38, 40]. Overall, the different Jun immunohistological expression patterns indicate differential involvement of Jun family members in TEC differentiation and can be paralleled with the different expression patterns of Jun family in epithelial cells of the normal human colorectal mucosa (no detectable expression of JunB, expression of c-Jun in scattered epithelial cells, and diffuse and strong expression of JunD) and the normal human oral mucosa (nil or low expression of c-Jun, JunB, and JunD) [43, 44].

In the present study, JunD was expressed by a sizeable proportion of thymocytes whereas p-c-Jun and JunB were detected in a few thymocytes. These different expression patterns indicate differential involvement of Jun family members in thymocyte differentiation. Our observations are consistent with Northern Blot findings showing that thymocytes express a high and constant level of JunD mRNA (in untreated thymocytes cultures at 0, 0.5, 1, 2, 4, and 6 h) whereas only a transient increase of c-Jun and JunB mRNA was observed between 0.5 and 1 h in these cultures [12]. Moreover, our observations are in agreement with immunohistological findings in other tissues of the immune system showing that c-Jun and JunB are weakly expressed in rare lymphoid cells in the germinal centers of tonsils [45] and JunB is faintly expressed in rare germinal center cells and small lymphocytes of reactive lymph nodes [46]. Furthermore, normal mature CD4 positive mouse splenic T-cells constitutively express JunD and JunD transgenic mice exhibit profound defects in the lymphoid system, such as altered T-cell proliferation and T-helper (Th) cell differentiation [47]. Collectively, the aforementioned findings provide immunohistological evidence that JunD may have a notable implication in the physiology of lymphoid T-cells. We also found that the expression of JunD was higher in medullary than cortical thymocytes. In view of evidence that JunD may positively or negatively regulate cell proliferation in a context-dependent manner [2, 4, 47], our findings suggest that JunD may be a negative regulator of thymocyte proliferation since these cells exhibit notably lower proliferation profile (determined by immunostaining with Ki67 and cyclins A, B1, D3, and E) in the medulla compared to the cortex [38, 40, 48].

Besides cell proliferation, the increase of JunD expression from the cortical to medullary thymocytes (present study) might also be implicated in the decrease of apoptosis from the cortical to medullary thymocytes [38, 40, 49] since JunD may have antiapoptotic activity [2, 4, 5]. However, a study in mice showed that overexpression of JunD cannot protect thymocytes from numerous apoptotic stimuli such as Fas (anti-CD95), anti-CD3, TNF-alpha, or UV-irradiation [47]. Nevertheless, JunD might be a positive regulator of thymocyte and TEC survival since JunD can protect cells from oxidative stress [2, 4].

In the present study, p-c-Jun or JunD expression was observed in a few CD11c positive thymic dendritic cells. This can be paralleled with previous immunohistological findings showing p-c-Jun expression in some follicular dendritic cells of reactive lymph nodes [50]. Moreover, there is evidence that JNK signaling, which is involved in c-Jun and JunD phosphorylation [2, 4], is activated during human monocyte-derived dendritic cell maturation [51]. Furthermore, AP-1 composed of JunD and Fra2 proteins was found to play a primary role in enhancing the transcription level of the CD11c gene in mouse dendritic cells [52]. Collectively, these findings suggest that c-Jun and JunD may be involved in the physiology of dendritic cells localized in various tissues including thymic dendritic cells.

In the present study, p-STAT3 expression was detected in a sizeable proportion of TEC with higher expression in cortical compared to medullary TEC including HB and the expression levels of p-STAT5 and p-STAT6 were low in TEC including HB. These findings indicate that the phosphorylated (activated) STAT, p-STAT3, p-STAT5, and p-STAT6, are differentially involved in TEC differentiation and are consistent with previous immunohistological findings showing distinct topographical distribution of STAT proteins in thymus. Indeed, STAT1, STAT2, and STAT6 were abundantly expressed in the entire HB whereas STAT5 was expressed in the outer layer of many HB [29]. Our findings indicate that activation of STAT5 and STAT6 occurs in a few TEC since p-STAT5 and p-STAT6 expressing TEC were less numerous than STAT5 and p-STAT6 positive ones [29]. The different expression patterns of p-STAT3, p-STAT5, and p-STAT6 in TEC (present study) may be related to different activation processes induced by various cytokines acting via the JAK-STAT pathway since TEC express various cytokine receptors [7, 8, 22, 24]. For example, STAT3 is activated by the cytokines IL-2, IL-6, and IL-10, STAT5 is activated by IL-2, IL-3, IL-5, IL-7, IL-9, and IL-15, and STAT6 is activated by IL-4 and IL-13 [8, 10, 53, 54]. Interestingly, there are lines of experimental evidence implicating activation of STAT1 and STAT3 in TEC differentiation. Indeed, proper medullary TEC maturation relies on coordinated cross-talk between multiple ligand-receptor complexes such as CD40-CD40L and receptor activating NF-*κ*B- (RANK-) RANK ligand (RANKL) [21, 22, 24] and* in vitro* RANK stimulation of medullary TEC progenitors results in the upregulation of various interferon-stimulated genes via a mechanism involving phosphorylation of STAT1 [55]. Moreover, downstream elements of fibroblast growth factor- (FGF-) mediated signaling, which is involved in TEC differentiation, may include STAT1 and STAT3 [17]. On the other hand, STAT activation in TEC might also affect thymocyte survival. Indeed, epithelium-specific STAT3-disrupted (STAT3−/−) mice showed severe depletion of TEC and subsequent thymocyte apoptosis [13]. It is, therefore, plausible that constitutive STAT3 activation in normal TEC may promote thymocyte survival possibly under the action of thymic hormones or cytokines [21, 22, 24]. Furthermore, STAT3 might also cooperate with c-Jun to affect TEC survival since a sizeable proportion of these cells express p-STAT3 and p-c-Jun with rather overlapping immunotopography in the cortex (present study) and there is* in vitro* evidence that cooperation between STAT3 and c-Jun suppresses Fas/CD95 transcription presumably interfering with cell apoptosis [6].

In previous immunohistological studies, phosphotyrosine-containing proteins were found to be present in high amounts in HB and p59-fyn and p60c-src which are members of the src family protein tyrosine kinases were also detected in TEC including HB [28]. Since STAT were reported to be associated with tyrosine phosphorylation signaling via the src family protein tyrosine kinases, the expression of various elements of the JAK-STAT pathway in HB [29] and the expression of p-STAT3, p-STAT5, and p-STAT6in TEC including HB (present study) imply that the two tyrosine phosphorylation signaling pathways may cross talk in TEC differentiation.

Immunohistological studies on normal thymus may also provide information for the histogenesis and the biology of thymic epithelial tumors (TET). For example, our previous findings that Bcl-2 is preferentially expressed in normal medullary TEC and thymomas while p53 is preferentially expressed in normal cortical TEC and thymomas [56] may provide information for the understanding of the histogenetic classification and the corticomedullary differentiation of TET [57, 58]. Moreover, compared with the normal thymic tissue, TET demonstrated higher expression of c-Jun and STAT3 and progressive TET harboring higher tumor stages exhibited higher expression of c-Jun and STAT3 [32, 33]. Therefore, further multiparametric immunohistological studies determining the expression patterns of the AP-1 and STAT proteins in both normal and neoplastic thymus might be useful to gain further insight in the pathogenesis of TET.

## 5. Conclusions

We used double immunostaining with markers of epithelial cells, lymphoid cells, dendritic cells, and macrophages to investigate the immunotopographical distribution and the expression levels of p-c-Jun, JunB, JunD, p-STAT3, p-STAT5, and p-STAT6 in normal postnatal human thymus. The major findings of the present study were the following: (a) JunD was expressed by a sizeable proportion of thymocytes with higher expression levels in medullary compared to cortical thymocytes, (b) p-c-Jun was expressed by a sizeable proportion of cortical and medullary TEC and HB, and (c) p-STAT3 was expressed by a sizeable proportion of TEC with higher expression levels in cortical compared to medullary TEC and HB. Furthermore, JunB and JunD were expressed in a few cortical TEC with higher expression levels in medullary TEC. p-c-Jun, JunB, p-STAT3, p-STAT5, and p-STAT6 were expressed by a few thymocytes. p-STAT5 and p-STAT6 were expressed by a few cortical and medullary TEC. Double immunostaining revealed p-c-Jun and JunD expression in rare CD11c positive dendritic cells. Collectively, our findings suggest a notable implication of JunD in the physiology of thymocytes and p-c-Jun and p-STAT3 in the physiology of TEC. Furthermore, the diversity of the immunotopographical distribution and the expression levels of p-c-Jun, JunB, JunD, p-STAT3, p-STAT5, and p-STAT6 in thymic cells indicates that the expression of p-c-Jun, JunB, JunD, p-STAT3, p-STAT5, and p-STAT6 is tightly regulated during thymic cell differentiation and they are differentially involved in the differentiation of TEC, thymocytes, and dendritic cells.

## Figures and Tables

**Figure 1:**
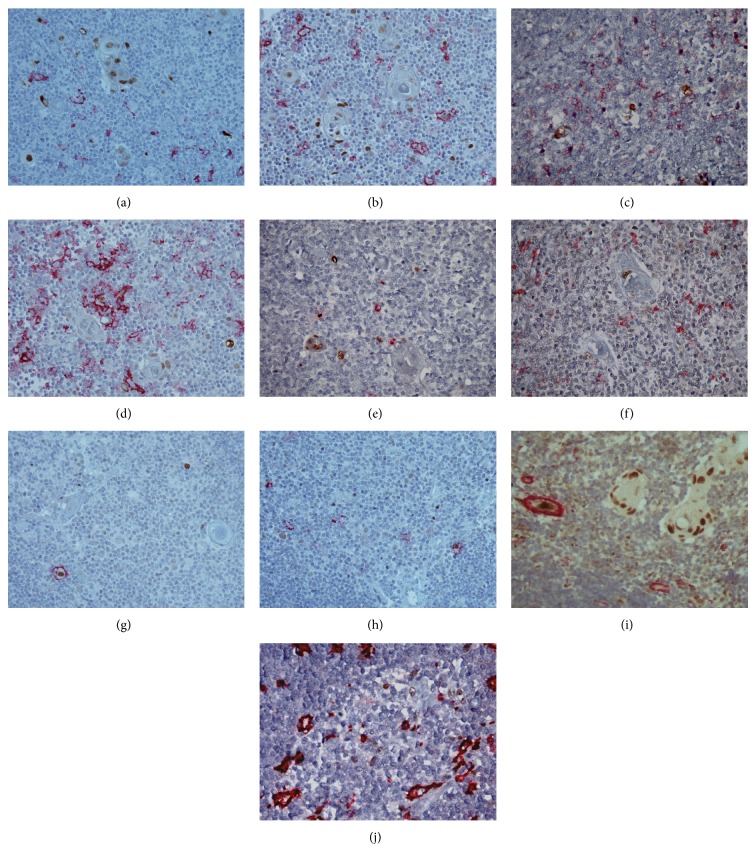
Immunohistological analysis of thymus using the double streptavidin-biotin peroxidase-labeled (brown staining)/alkaline phosphatase and antialkaline phosphatase (red staining) immunohistochemical procedures. Double immunostaining for (a) p-c-Jun (brown)/CD163 (red) (magnification ×400), (b) p-c-Jun (brown)/CD11c (red) (magnification ×400), (c) p-c-Jun (brown)/CD123 (red) (magnification ×400), (d) p-c-Jun (brown)/CD11c (red) (magnification ×400), (e) p-c-Jun (brown)/chromogranin (red) (magnification ×400), (f) JunD (brown)/CD163 (red) (magnification ×400), (g) JunD (brown)/CD11c (red) (magnification ×400), (h) JunD (brown)/CD11c (red) (magnification ×400), (i) JunD (brown)/smooth muscle actin (red) (magnification ×400), and (j) p-STAT3/CD163 (red) (magnification ×600).

**Figure 2:**
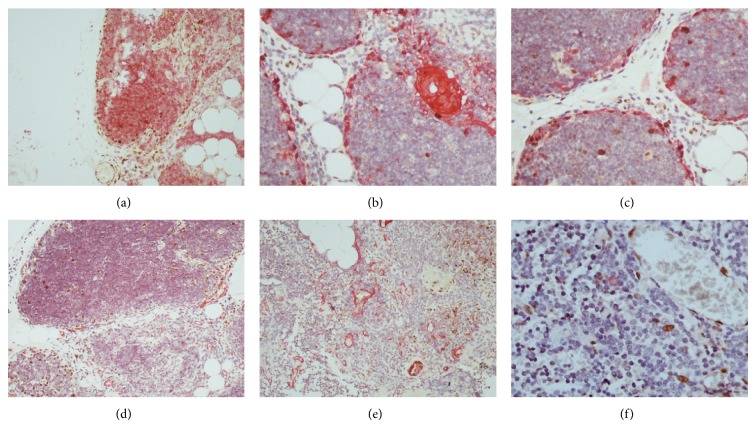
Immunohistological analysis of thymus using the double streptavidin-biotin peroxidase-labeled (brown staining)/alkaline phosphatase and antialkaline phosphatase (red staining) immunohistochemical procedures. Double immunostaining for (a) p-c-Jun (brown)/CD3 (red) (magnification ×200), (b) p-c-Jun (brown)/pancytokeratin (red) (magnification ×400), (c) p-c-Jun (brown)/pancytokeratin (red) (magnification ×400), (d) p-c-Jun (brown)/smooth muscle actin (red) (magnification ×200), (e) p-c-Jun (brown)/smooth muscle actin (red) (magnification ×200), and (f) p-STAT3 (brown)/CD123 (red) (magnification ×600).

**Table 1:** Expression of p-c-Jun, JunB, JunD, p-STAT3, p-STAT5, and p-STAT6 in thymocytes.

	Cortical	Medullary
p-c-Jun	−/+	−/+
JunB	−/+	−/+
JunD	+/−	++
p-STAT3	−/+	−/+
p-STAT5	−/+	−/+
p-STAT6	−/+	−/+

<1% (−/+); 1–10% (+/−); 10–20% (+); 20%–50% (++).

**Table 2:** Expression of p-c-Jun, JunB, JunD, p-STAT3, p-STAT5, and p-STAT6 in thymic epithelial cells.

	Cortical	Medullary	Hassall bodies
p-c-Jun	++	++	++
JunB	−/+	+/−	+/−
JunD	−/+	+	+
p-STAT3	++	−/+	−/+
p-STAT5	−/+	−/+	−/+
p-STAT6	−/+	−/+	−/+

<1% (−/+); 1–10% (+/−); 10–20% (+); 20%–50% (++).
